# Toward a Greener World—Cyclodextrin Derivatization by Mechanochemistry

**DOI:** 10.3390/molecules26175193

**Published:** 2021-08-27

**Authors:** László Jicsinszky, Giancarlo Cravotto

**Affiliations:** Department of Drug Science and Technology, University of Turin, Via P. Giuria 9, 10125 Turin, Italy

**Keywords:** solvent-free reactions, ball mill, synthesis, high yields, sustainable production, ultrasound, complex

## Abstract

Cyclodextrin (CD) derivatives are a challenge, mainly due to solubility problems. In many cases, the synthesis of CD derivatives requires high-boiling solvents, whereas the product isolation from the aqueous methods often requires energy-intensive processes. Complex formation faces similar challenges in that it involves interacting materials with conflicting properties. However, many authors also refer to the formation of non-covalent bonds, such as the formation of inclusion complexes or metal–organic networks, as reactions or synthesis, which makes it difficult to classify the technical papers. In many cases, the solubility of both the starting material and the product in the same solvent differs significantly. The sweetest point of mechanochemistry is the reduced demand or complete elimination of solvents from the synthesis. The lack of solvents can make syntheses more economical and greener. The limited molecular movements in solid-state allow the preparation of CD derivatives, which are difficult to produce under solvent reaction conditions. A mechanochemical reaction generally has a higher reagent utilization rate. When the reaction yields a good guest co-product, solvent-free conditions can be slower than in solution conditions. Regioselective syntheses of per-6-amino and alkylthio-CD derivatives or insoluble cyclodextrin polymers and nanosponges are good examples of what a greener technology can offer through solvent-free reaction conditions. In the case of thiolated CD derivatives, the absence of solvents results in significant suppression of the thiol group oxidation, too. The insoluble polymer synthesis is also more efficient when using the same molar ratio of the reagents as the solution reaction. Solid reactants not only reduce the chance of hydrolysis of multifunctional reactants or side reactions, but the spatial proximity of macrocycles also reduces the length of the spacing formed by the crosslinker. The structure of insoluble polymers of the mechanochemical reactions generally is more compact, with fewer and shorter hydrophilic arms than the products of the solution reactions.

## 1. Introduction

The fabrication of cyclodextrins (CDs) is essentially a green process in which the industrial waste can further utilize in various other procedures, such as industrial alcohol production. Although mass production of CDs is essentially a green process, the same can hardly be true for CD derivatization in general. The most used CD derivatives, such as (2-hydroxy)propyl- and 4-sulfobutyl-βCDs (HPβCD and SBβCD), are produced in a concentrated aqueous solution; the purification and, particularly, solidification are energy-intensive processes. From a synthetic point of view, due to limited solubility in volatile green organic solvents, the production of many CD derivatives does not meet the requirements of green chemistry. Neither does, usually, the preparation of CD complexes. Although water is an environment-compatible solvent, the energy demand of its removal can considerably increase the costs of manufacturing.

At the beginning of this century, the Twelve Principles of Green Engineering laid down the fundamental aspects in process design for a sustainable future [1]. Despite all efforts, and mainly due to the often conflicting requirements, it is not always possible to comply at the same time with all these directives in the chemical industry. In addition to new synthetic methods, the repurposing of some old large-scale applications can replace many energy-intensive, often polluting technologies. Although many inorganic chemical productions utilize mechanochemical techniques, the penetration of this technology to the organic syntheses is relatively new [2,3,4].

Mechanochemistry combines mechanical and chemical transformations at the molecular level and comprises chemical conversions triggered by physical and physicochemical processes. These transformations are milling, shearing, and sonochemical manipulations; utilization of hydrodynamic cavitation [5]; and tribology [6,7,8,9]. Many times the limit between these processes is not sharp, and the elementary steps are mixed. Mechanochemistry is principally the transfer of mechanical energy to the chemical activation of the reactant(s), with the chemical reactions either in the presence or lack of a liquid phase rarely restricted to a simple energy transfer. Technologically, some fundamental processes of mechanochemistry, such as changing the particle sizes by grinding, or influencing the shearing and tribologic behavior, have been utilized for thousands of years. Using the ultrasound (US) in laboratory practice, as the technology has allowed it, is a relatively old method; the imposing of the potential in hydrodynamic cavitation is still a curiosity and being continuously developed.

The green point of view can divide mechanochemical manipulations into two technologies: (a) reaction in solution and (b) reaction in solid- or quasi-solid state. The standard reaction media of sonochemical and hydrodynamic cavitation-induced reactions uses solutions, where the solvent is usually water. When the reaction products are not isolated, or if the manipulations aim to remove or degrade chemicals, e.g., in wastewater treatment, these technologies offer a green and energy-efficient alternative to conventional methods. Solid-state reactions use a minor part of mechanochemical technology; however, in general, they can offer a truly green approach when compared with traditional synthetic methods. In many cases, the reactant may also be partially liquid or gaseous, or it may become liquid/gaseous during the operation, i.e., these reactions are not always purely solid-state reactions. Liquefaction can be the result of either temperature or chemical reasons. On the other hand, there are cases where small changes in the same technology can change a mechanical transformation into a chemical reaction. In this mini-overview, we use mechanochemistry in a slightly broader sense and briefly summarize the processes otherwise used in mechanochemical treatments in complex preparation.

Although shear and tribological reactions can represent key chemical transformations, they are less suitable for manufacturing only, while acoustic or hydrodynamic cavitation and grinding methods provide green production tools. It is also true that shear and tribological transformation are involved in almost all mechanochemical processes.

The solid-state reactions are carried out characteristically in mills or mortars. A remarkable case of mechanochemical transformations is when the treatment uses a mortar and pestle. This manipulation, often erroneously called milling, can be used to induce chemical reactions, as well. Although mortar technology is the simplest and fastest way for mechanochemical activation, the transferred energy is limited and rarely enough to trigger chemical reactions. It is a practical tool for transformations that are little affected by the environment (light, oxygen, CO_2_, etc., or evaporation of components).

In recent decades, many new grinding technologies developed, and existing methods significantly improved. The theory of grinding was developed mainly by the mineral industry, principally for particle-size-reduction purposes. Although the literature gives many examples of experimental studies with different types of mills and with various mineral powders, there are still little data available on both host–guest complexation and their use in chemical reactions. Another limiting factor is that the mineral manufacturing industries developed approaches aimed at their needs, so relatively little experience has accumulated on the property changes of organics and pharmaceuticals during milling. The fundamental criteria include the ease of determining operating parameters and the simplicity of cleaning to remove contaminants, which is particularly important for multi-product installations. Another potentially limiting factor is the physical contamination of the ground materials, but fortunately, many practically non-degradable milling media are now available, and all the parameters of the grinding process can be tailor-made.

There are principally two main groups of grinding technology [10], within which there are, of course, further subgroups [11]. Jet mills use high-speed compressed air or inert gas jets to grind materials by colliding particles against each other and the wall. The compressed gas is forced into the mill through nozzles perpendicular to the cylinder wall, creating a vortex. The gas usually exits the mill through a tube along the cylinder axis, while the powdery product discharges at the bottom of the unit.

The other class is the impact mills, where the attrition or grinding mills grind materials by mechanical impact. Before the industrial revolution, grinding was mainly performed by attrition grinding or grinding the material between two surfaces, which is still the dominant comminution method for agricultural products today.

Gravity impact mills pulverize the material in a rotating chamber, and then the pulverized material is converted into finer particles by the repeated impact of larger pieces. These autogenous impact mills usually do not contain foreign material. The addition of balls (ball mills, BMs) or rods can intensify the crushing operation. There are several subclasses of gravity impact mills, commonly referred to as dynamic impact mills, because a dynamic method is applied to increase the energy efficiency of the impacts.

While jet technology is typically for pulverization purposes, impact mills are also suitable for homogenizing materials. From the point of view of chemical reactions, inorganic chemical syntheses exploited first the impact mills, and their use in organic chemistry spread only later, especially with the advent of vibrating and BMs with higher energy transfer. With the increasing number of publications describing applications in organic synthesis, the view that this method is only utilizable for reactions that proceed almost spontaneously when reactants are in close spatial proximity is becoming obsolete. Although this observation is more valid for manual manipulation in a mortar, it is less for syntheses in a BM.

Although the rotation speed of classical BMs is low, and thus the energy transfer is lower in a given time, these devices are also suitable for industrial-scale production. Vibratory, mixer, and planetary versions, often referred to as high-energy grinding technologies, are mainly advantageous for the high kinetic energy conversion to chemical activation. These technologies have enabled numerous new chemical syntheses, mainly in the solid phase, in recent decades. In this review, the main focus is on these technologies.

The laboratory practice widely uses US, and in the case of low-energy US irradiation, the principal purpose is to disintegrate particles or prepare solutions and emulsions. In these manipulations, the main goal is to reach smaller solid particles or larger contact surfaces. The laboratory cleaners are commonly used to accelerate the dissolution of solids, removing impurities from the glassware; initialize crystallization; or degas solutions, and are rarely used to trigger chemical reactions. The latter usually occurs when a poorly soluble reactant or a heterogeneous catalyst requires further physical destruction by the US. In these cases, the size of the reaction is limited, and moderate warming of the US-transmission medium is often sufficient, too. Although the US-assisted dissolution or cleaning is a part of the laboratory routine, only a few scientists and assistants connect the US to cavitation.

When the velocity of a flowing system suddenly increases, Bernoulli’s law states that the pressure decreases. Cavitation occurs when the absolute pressure somewhere in the flow decreases to the pressure of saturated water vapor at the prevailing temperature, and then the homogeneity of the flowing medium is lost, forming vapor bubbles inside the liquid. The bubbles are again at higher pressure, collapsing. The consequences are unwanted vibrations, oscillations, noise, and structural failures due to the collapse of bubbles under higher pressure. There are cases where the phenomenon can be exploited, such as forming emulsions, cleaning surfaces, cavitation pumping, or energy transfer for chemical reactions. Since violent collapse only occurs when the enlarged bubble is mostly vapor, this condition is unfavorable in applications where the aim is to induce cavitation. Triggering cavitation has two ways.

(a) Acoustic cavitation appears when ultrasound waves entering the liquid medium cause alternating frequency-dependent high- and low-pressure cycles. In the low-pressure period, the high-intensity ultrasonic waves create tiny vacuum bubbles in the liquid. When these bubbles reach a volume at which they can no longer absorb energy, they collapse violently in the high-pressure cycle. The collision can generate locally high temperatures and pressures. Cavitation bubble collision results in high-velocity liquid jets, too. The acoustic cavitation transfers about one order of magnitude less energy than hydrodynamic cavitation [12].

(b) Hydrodynamic cavitation can be created by passing a fluid through a channel constricted by a defined flow rate or by mechanical rotation in the liquid. In a constricted channel based on the specific geometry of the system, the combination of pressure and kinetic energy can create high-energy bubbles that result in hydrodynamic cavitation after the local constriction. Using ultra-turrax for dispersing or dissolving various materials is the laboratory utilization of rotating object-created cavitation.

The reaction mechanism in BMing, particularly when no liquid phase presents, significantly differs from conventional solution reactions [2,3,4,13,14,15]. However, the application of liquids that cannot solubilize the reaction components and rarely participate in the chemical transformations can influence the reaction energetics. The wet-milling technologies use minimal amounts of solvents, and the reaction characteristics are still solid–solid reactions. In many cases, water formation can significantly affect the consistency of the milled materials. In general, the water increases the density of the solids by sticking the particles stick together, resulting in a hardly crackable solid. This unwanted phenomenon can restrict the movement of the reacting particles and can reduce the reaction speed. The shearing forces can disrupt the formed hard solids, which finally revitalize the reaction.

All physical treatments transfer energies to the medium, which, depending on the conditions, can accelerate the targeted reaction or initialize decomposition. Thermal changes in solutions are relatively easy to control on both laboratory and industrial scales. Although both heat and mass transfer are limited in solids, this feature offers many exploitable advantages. The thermal effect depends on the grinding conditions, and under milling conditions, this is not unusual, and local overheating often occurs within the reactor because the mass of the colliding balls is much greater than the mass of the particles entrapped between them. Despite the intensive energy transfer, due to the limited heat transfer, the bulk temperature can remain below 100–120 °C [16,17,18].

Common reaction vessels are usually not transparent, and this prevents visual control. When a liquid phase appears during milling, the reaction mechanisms may become more complex and mix with solution mechanisms. A solid-state reaction setup is usually significantly easier than conventional cases, but the absence of solvents can also reduce the time and energy required for workup and isolation. Many grinding media are available, but not all are suitable for all types of reactions. The choice of the best grinding media depends on the chemical and physical properties of both the reactants and the products and often influences the reaction efficacy. The vast and long experience in mineral processing can also help to select the most suitable conditions.

In this review, because substantially only physical processes are involved in many mechanochemical methods of complex preparations, the complexation methods are superficially discussed.

## 2. Discussion

### 2.1. Complexes

**Figure 1:**
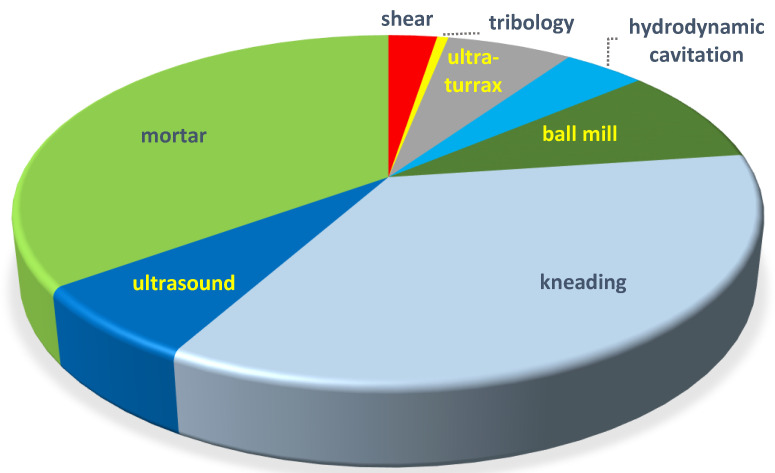
Distribution of various complexations in the technical papers till 28 July 2021. Due to the overlaps of some topics, such as grinding and kneading, hydrodynamic cavitation/ultra-turrax, and ultrasonic mixing, the percentages carry little information.

The publication distribution of mechanochemical methods used in the preparation of CD complexes is seen in Figure 1.

#### 2.1.1. Complexation in Mortar

Mechanical agitations are usually complicated processes, particularly in complex preparations, and cannot always describe one methodology.

The mixing of solids in a mortar is a complex mechanical process, crushing mixed with shearing, and often tribological effects also playing a significant role. Simple mixing of solid components rarely results in complexation, but the addition of some water or organic solvents transforms the process into kneading, which is another frequent CD complexation method. Although many papers discuss complexes prepared by kneading, the publications often contain a typical theoretical error, namely that if the components were mixed in a 1:1 molar ratio, the composition of the resulting complex is 1:1. The complex formation is an equilibrium process, and one or both of the components may dissolve in the solvent used. The finally obtained solid is a mixture of complex(es) and free species. Though it is possible to induce a mechanochemical reaction in a mortar, this manipulation does not usually provide sufficient mechanical energy to break or form chemical bonds. In the case of complexes prepared by kneading, chemical reactions are unlikely to occur, except for hydrolysis at extreme pH, photochemical oxidation of sensitive compounds from the open system, or carbonate formation due to the presence of oxygen in air or CO_2_. These reactions can usually occur without mortar treatment for compounds that are sensitive to environmental influences. The mechanochemical points of mortar grinding are discussed in a later section.

#### 2.1.2. Complexation in Mills

Although milling technologies have been around for thousands of years, they have changed more than ever in the last century. Whereas in the past the industrialization was characterized primarily by the increase in size, milling technologies have changed significantly in recent decades, both theoretical and materials terms, allowing much more energy-efficient processes to be designed and applied. In contrast to mills, crushers are used primarily in comminutions, and homogenization of various materials is usually a secondary target. Grinding combines both the comminution and homogenization tasks. The use of milling technology in solid complex production or targeted selective extraction is relatively new. With the development of mechanically more resistant milling media materials, such as agate or zirconium oxide (ZrO_2_), the fundamental problem of grinding technology, namely contamination due to mechanical wear of the grinding media, has been solved. Intensive developments have not only made complex preparation on an industrial scale possible, but the methodology has also become applicable to the pharmaceutical industry. The milling process can be quite complex and lead to physical transformations that are difficult to describe. At first, the phase transformations in grinding of an aspirin/DIMEB complex, and although the ground complex has a better solubility profile, the chemical stability of aspirin was reduced [19].

Naproxen/βCD complex prepared in a ceramic BM showed a significantly better solubility profile than the crystalline version [20]. The similarly produced complex of amphiphilic carboxymethylated ethyl-βCD and diltiazem showed excellent controlled release in an acidic aqueous solution [21]. Almost ten years later, the shearing force effect studies on the physicochemical properties of ibuprofen/βCD complexes in a roll mill was a significant step ahead for the industrialization of complex preparation in solid-state [22]. The first movement toward the use of high-energy BMing studied the phase transitions of permethylated βCD in a vibration mill [23]. The phase transitions in another high-energy milling in a planetary BM revealed that the glass transition of the amorphized solid occurs above the thermal degradation point [24]. Milling-condition-optimization studies on steroid/βCD complexes in a planetary BM showed 200–300 rpm optimal evolution speed [25]. Since the planetary mill used has a sun-wheel-to-jar ratio of 1:2, the results suggest that the method is transferable to roll-mills, since the maximum speed of laboratory mills is about 400 rpm. Although this is still far from the 20–50 rpm of pilot plant/industrial mills and the limited number of experiments does not allow generalization, the production of drug/CD complexes in kilogram sizes seems feasible with laboratory roller mills.

**Figure 2:**
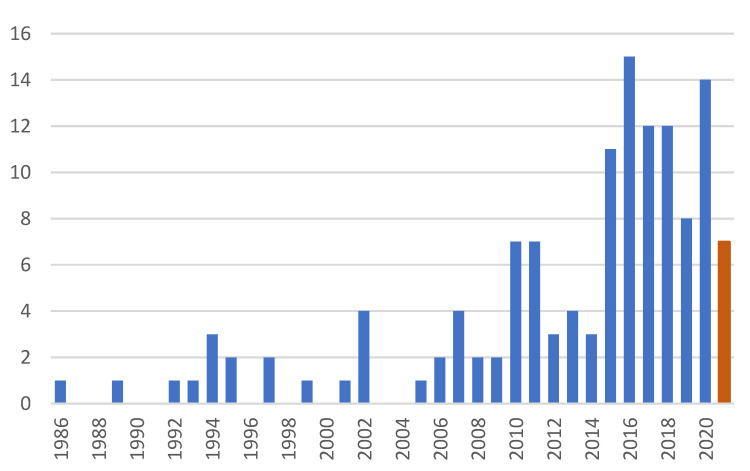
Number of publications on the application of BM in CD complex preparations till 28 July 2021.

Since the first application of BM in the CD complex preparation, more than 100 technical papers have appeared as the publication dynamics seen in Figure 2, demonstrating the slow penetration of the grinding technologies into everyday practice. A recent review [26] well-summarizes the various aspects from the amorphization to the preparation of ground CD complexes, and as a general conclusion, the ground complexes have improved physicochemical properties.

#### 2.1.3. Complexation with Kneading

The kneading preparation of CD complexes is among the oldest methods, and usually, it is carried out in mortar by hand. Technically, this is a slight variation of wet grinding, where the wetting agent is usually water or EtOH; however, it is not a literal mechanochemical process. Energy transfer is both moderate and low efficiency, with prolonged hand kneading through the viscous mass resulting in personally perceptible energy transfer. This method targets the structural changes by secondary forces, such as H-bonds, conformational energies, or hydrophobic interactions [27,28]. Even though this method uses an open system and is difficult to reproduce due to manual manipulation, many publications report that this method belongs to one of the best complex preparation methods. However, it is also true that many times the descriptions are superficial for correct reproduction. In the early days of the widespread investigations of CD-complexes, a systematic study revealed the importance of choosing the appropriate CD/water and guest/water ratios [29]. Freeze-drying of a homogeneous solution of the ketoprofen/SBβCD complex was more efficient than kneading in producing the solid complex; however, the host/guest ratios for both methods resulted in similar trends in the physicochemical properties of the solids [30]. However, the natural βCD and oleoresin complexation worked oppositely [31]. Most of the publications deal with comparisons of the complexation efficiency by the various complex preparation methods, but a general conclusion is difficult to state [32,33,34].

#### 2.1.4. Complexation with Ultra-Turrax, a Transition to Hydrodynamic Cavitation

The high-speed stirring of solid/liquid or liquid/liquid systems is a daily laboratory homogenization method. The operating speed of the ultra-turrax is 15,000–30,000 rpm, and although the conditions are theoretically suitable for cavitation effects, this rarely occurs. Therefore, although complexation articles mention this method, the high-speed mixing is not associated with the development of mechanochemical activation conditions and cavitation effects. Primarily, in these cases, the dissolved gas content of the liquid may be high, partly due to the design of the device and partly due to the lack of a system to remove dissolved gases from the system. The initial nucleus size where the cavitation phenomenon can start at about 100 μm. Bubbles of this size can exist in a liquid in the absence of cavitation if the dissolved gas content of the liquid is high. Therefore, in general, the ultra-turrax-induced cavitation is transient and short-lived, and thus this phenomenon is not significant, unlike ultrasonic homogenization. Therefore, and due to the incomplete literature data, we omit further discussion.

#### 2.1.5. Spray-Drying, a Transition to Hydrodynamic Cavitation

Spray-drying is a mild and large-scale applicable process for solvent—dominantly water—removal. Many factors on both the inlet and outlet sides influence the particle size of the obtained solids. Although hydrodynamic cavitation can further reduce the particle size produced during spray drying, this new technology has not been included yet in the toolbox of solidification of CD complexes. However, spray-drying, similar to freeze-drying, is mainly used to remove solvents from homogeneous solutions of complexes [35,36].

Another new technology in spray-drying, namely electro-spinning, uses an electric field for producing polymeric nanomaterials in spray-drying [36]. The method combines many different micronization techniques for further size reduction of the solution droplets [37]. Although the spray-drying technology is not new, the connection with mechanochemistry is not always clear.

#### 2.1.6. Complexation with Hydrodynamic Cavitation

The effect of hydrodynamic cavitation on the non-covalent associations, particularly by the short-term temperature and pressure shocks, needs further studies, and the currently available information in the CD complexes is limited [37,38].

The homogenization process using Tween 80 and Span 80 surfactants was energetically more effective by hydrodynamic cavitation than by the US [39]. CD complexation in insect-repellent formulations can exploit the potential of the hydrodynamic cavitation process [40].

Supercritical assisted spray-drying has recently become one of the most efficient techniques for the production of nanoparticles. This process prepares the mix of supercritical CO_2_ and a complex solution, and then this mixture is injected into the cyclone at near atmospheric pressure. Since the surface tension of the expanded solution is almost zero, together with the solution’s low viscosity, the formation of a solid complex in the solution is limited, which improves the efficacy of the sputtering process. A hydrodynamic cavitation mixer can further enhance this effect by increasing mass transfer, which is beneficial for solidifying complexes of thermosensitive materials [41].

#### 2.1.7. Complexation with Ultrasound

Another method to trigger cavitation effects is the US irradiations [38]. Although the sonication of suspensions and emulsions is a common practice, the manipulation details are rarely published. There are many US laboratory cleaners on the market, but not all of them provide the same wavelength and energy. Comparisons of probe (horn) type and bath methods are even less frequently published. This type of comparison would be necessary for method transfer from experimental laboratory scale to kilolab or pilot plant operation. Although the probe version seems more efficient in particle disintegration and possibly also in complex formation, the available information is limited to draw a general conclusion [42,43].

Preparation of βCD templated CdSe spherical hollow quantum dots used 75 W/cm^2^ flux of 20 kHz US [44,45,46]. The formed uniform solid showed electrochemical luminescence in sensor applications. Hexavalent chromium ion, Cr(VI), is a carcinogen metal ion, and its quantitative analysis is mandatory in many products, workplaces, or in the environment as ground and potable water or wastewater plants. A colorimetric method development complexed βCD with functionalized gold–iron nanoparticles (βCD/Au-FeNPs), using high-energy US irradiation, which reduced the spectrophotometric limit of detection to the 50 nM level [47]. The higher sensitivity can be associated with the clustering of chromate ions with βCD/Au-FeNPs complexes.

Concentration, frequency, and DS dependency of (2-hydroxy)propyl βCD [42] (HPβCD) templated preparation of 1-hexyl-3-methylimidazolium tetraphenylborate nano-assemblies studies showed significantly smaller particle sizes (hydrodynamic radius) in comparison with the non-templated method. Although the microwave irradiation further reduced the particle size of salts produced by non-template sonication (55 and 20 kHz), the average hydrodynamic radii were still 5 to 6 times larger than in the presence of HPβCD. Zeta-potential differences showed no concentration and frequency dependence.

βCD, HPβCD, and 4-sulfobutylated βCD (SBβCD) could effectively complex a nonsteroidal anti-inflammatory drug, salicyl salicylate (salsalate), using 60 W of 24 kHz sonication [48]. The preparation of HPβCD complexes of cumin aldehyde [49] and isoeugenol [50] used 60 W horn-type 20 kHz US irradiation and was significantly more effective than classic methods of the native form of host molecules. Resveratrol is a naturally occurring pharmacologically active and light- and oxygen-sensitive antioxidant. A nanoemulsion of resveratrol/HPβCD prepared by acoustic cavitation resulted in 20–30 nm particles with higher encapsulation efficiency than other attempts with ≈10% resveratrol content. The aqueous solution of the formed composite was almost transparent after 7 min US treatment both in batch (40 kHz, 450 W) and probe (26 kHz, 60 W) sonication [51]. Bioactive compounds of various physical forms of olive pomace, using βCD and US (40 kHz, 100 W), significantly increased the total phenolic content of the aqueous solution [52].

Imidazolinone herbicides are popular herbicides in soybean and other legume plants to control different grasses and broadleaf weeds. These herbicides have phytotoxic effects on various plants such as cotton, rape, potatoes, etc. The pH of soils and the organic and clay content significantly influence the adsorption and persistence of the imidazolinone herbicides, and their cheap and effective removal would be desirable. With 30 min of 40 kHz sonication, various chitosan/βCD supramolecular associates were prepared, and their biocomposites were suitable for the decontamination of Indian soil samples [53].

The degradation of the CD complex is going in the opposite direction to the traditional ones, and as CDs are becoming utilized for the recovery of certain valuable biological materials, these CD-recovery technologies will come to the fore soon. An example is the cholesterol removal of foods. Although the method was patented more than a half-century ago, the applied benzene and hexane are not food-friendly organic solvents [54]. Twenty years later, as CDs became more accessible and economical, the production of cholesterol-free foods became safer, and slowly an entire industry developed for this methodology [55,56]. Green production requires a green regeneration process of cholesterol and βCD also, instead of their wasting. Usually, due to solubility issues of cholesterol, the regeneration of CD from the complex requires low-polarity solvents. The solid/liquid extraction methodology of cholesterol complex decomposition requires the safe handling of organic solvents and high energy demand. The food industry is the largest cholesterol CD complex producing segment, and among other continuously developed methods, a US-assisted extraction procedure showed the highest recovery ratio compared to reflux and Soxhlet extractions. Energy efficiency point of view in a 10% EtOH suspension, using 40 kHz US and 0.49 W/cm^3^ (250 W input power) acoustic energy density, showed the best result [43].

The first well-characterized 4,6-benzylidene glucosamine catalyst prepared by acoustic cavitation used 26 kHz and 200 W probe sonication to achieve a soluble complex with βCD [57].

The sonication-generated microturbulence in organic liquid/water mixtures results in fine emulsion that intensifies the interphase transport of a contaminant of liquid fuels, dibenzothiophene. The radicals produced by transient cavitation oxidize dibenzothiophene to sulfone and sulfoxide, which also enhances microbial biodesulfurization. Transport of substrate and product across the cell wall followed Michaelis–Menten kinetics. Due to high shear and cavitation induced by ultrasound, the presence of βCD negatively influenced the dibenzothiophene transport [58].

#### 2.1.8. Complexes under Shearing

In systems with solid particles, the shearing forces occur whenever the particles collide, and fragmentation occurs as a result. It is difficult to separate these forces in individual mechanochemical processes. Although there are currently a few publications that show that shear forces alone influence the complex formation, various CD complexes can exploit these forces. Shear forces are also present in liquids, because adjacent layers of the fluid move with different velocities compared to each other. As Newton’s law of viscosity defines [59], in a laminar flow, the shear stress between adjacent flowing layers is proportional to the velocity gradients between the two layers. The energy in fluids is usually insufficient to utilize for complex preparations, but the reverse effect, i.e., the decomposition of complexes by shear forces, is more common.

Preparation of α-mangostin complex with (2-hydroxy-3-*N*,*N*,*N*-trimethylamino)propyl βCD grafted-chitosan used a high-speed homogenizer [60]. The α-mangostin release kinetics of the complex at various speeds in simulated saliva showed encapsulation efficiency dependency.

The combination of Fe_3_O_4_ nanoparticles and βCD-based ethanolamine-functionalized poly(glycidyl methacrylate) showed anticancer efficacy by the magnetic nanoparticle accumulation in tumor cells. The alternating magnetic field can generate a high shear force that destroys tumor cells [61].

In many cases, complexes can increase the shear strength of the formulation, but the role of CDs or their complexes is not always clear. CD maleate, copolymerized with acrylamide and 2-acrylamide-2-methylpropanesulfonic acid, as a silica gel [62] or montmorillonite hybrid [63], showed significantly higher physical stability and shear strength compared to the organic polymer alone. A betaine/cyclodextrin hyperbranched copolymer showed similar favorable property changes [64,65]. βCD-modified alginate and a methacrylated gelatin enabled the design of a shear-thinnable hydrogel that disintegrates under the shear forces applied during injection and becomes self-healing after they cease [66]. A combination of αCD/nonanyl modified poly(vinyl alcohol) with α-tricalcium phosphate showed excellent adhesion properties with dental implants. The complex exhibits thixotropic properties and provides significantly higher bonding and shear adhesion than the titanium plate-based commercial products [67]. Supramolecular αCD/4-PEG hydrogels also exhibit thixotropic properties, and they form shear stress-controlled reversible gel-solution transition. From these hydrogels, the release kinetics of a drug, such as the glaucoma drug brimonidine, can be controlled by shear stress, making them suitable for the preparation of injectable drug formulations [68]. The αCD content can govern both the shearing dynamics and strength properties of a methoxy polyethylene glycol conjugated arginine-functionalized poly(l-lysine)dendron complex. The formed hydrogel was suitable for a tailored shRNA plasmid gene therapy [69]. Another αCD content controlled shearing properties of a hydrogel complex with a glycol chitosan-Pluronic F127 conjugate and doxorubicin showed good local cell-targeting properties for chemotherapy [70]. The γCD can regulate the drug-release enantiopreferences by shear forces in sodium deoxycholate/TRIS hydrogels [71]. Polyrotaxanes, prepared from αCD and PEG-2000, also showed CD-content-dependent shearing properties in DMSO [72]. Three-dimensional bioprinting technology offers a promising strategy for the production of artificial tissues and organs. To this end, the main goal of the bioprinting process is to produce a bioink with ideal mechanical properties, without sacrificing biocompatibility; thus, PEG/chitosan, αCD, and gelatin-based hydrogel-based bioinks seem suitable. Aggregation of pseudopolyrotaxane-like side chains formed by host–substrate interactions between αCD and PEG side chains causes structural changes in the pre-crosslinked hydrogel under shear forces [73].

Hydrophobic ethoxylated urethane is another type of hydrophobically modified polyethylene glycol that can form a temporary network structure in water. Methylated βCD in the solution can control the effective elastic chain density and relaxation time and can potentially be used to release various drug molecules [74].

#### 2.1.9. Tribology of Complexes

Although tribology is less suitable for complex preparations, it can utilize complexes to improve the tribological properties of materials or protect the used materials from various chemical decompositions.

Possible applications for polystyrene/poly(dimethylsiloxane) blends include hydrophobic surfaces, membranes, and tribology [75,76]. The chemically dissimilar polymers generally have positive enthalpy and minimal entropy of mixing, so they are immiscible. Improving the mixing and stabilization of incompatible blends can usually be achieved in several ways: reactive compatibilization physically binds the blended polymer in a crosslinked microstructure or using a compatibilizer—a block copolymer consisting of chemically identical blocks to the homopolymers in the blend. The compatibilizer reduces the interfacial energy at the interface between the two phases, resulting in a tighter mixing. The γ-cyclodextrin core and polystyrene arms as compatibilizers have developed with several advantages over conventional block copolymer compatibilizers. Many different polymers are compatible with the CD core, which means that the same CD-star molecule is applicable for different polymer blends. In addition, the diameter of the CDs limits the polymers that can fit into the cavity, which also limits the complex formation, and the different cavity diameters of CDs are suitable to incorporate the desired polymers selectively. Since CD-star blends have a very homogeneous morphology, blends without CD-star resulted in a high degree of phase separation. The CD-star/poly(dimethylsiloxane) films tested exhibited significantly different thermal and mechanical properties with improved retention of poly(dimethylsiloxane). About twelve of the 24 γ-CD hydroxyl groups were involved in the polystyrene arms formation, using a brominated initiator for radical atom transfer polymerization [77]. Although the presented preparations for CD-star polymers and composites are far from green chemistry processes, the prepared materials can lessen the ambient impact of tribological aids by reducing the amounts of environmentally incompatible technical materials.

### 2.2. Chemical Transformations

**Figure 3:**
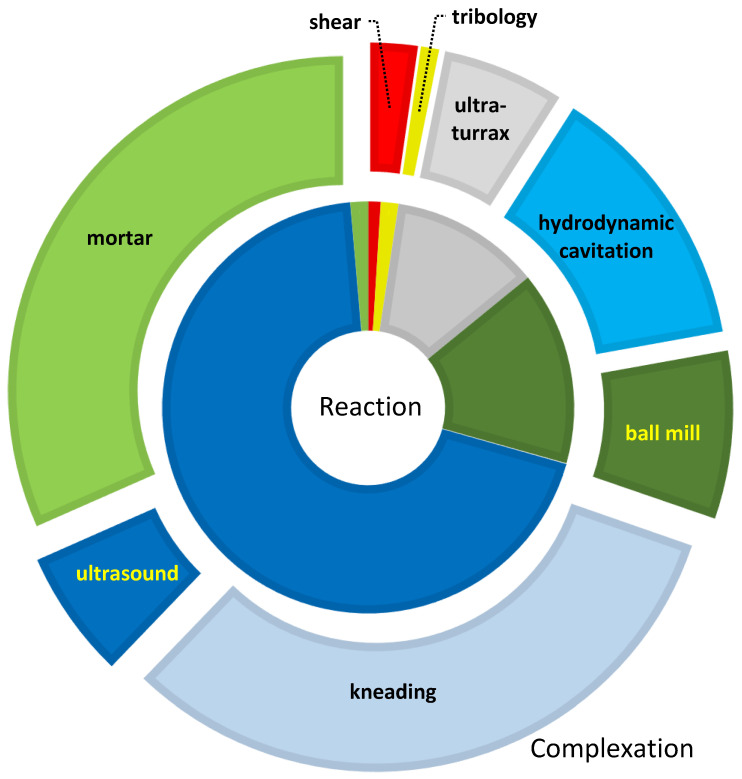
Participation of various mechanochemical methods in chemical transformations (inner slices) and complex preparations (outer arcs) in technical papers till 28 July 2021. Due to the overlaps of some topics, such as grinding and kneading, hydrodynamic cavitation/ultra-turrax, and ultrasonic mixing, the percentages carry little information.

While solution reactions take a smaller share of the complexation, they are more dominant in mechanochemical manipulations, as shown in Figure 3, while the simplest milling method virtually disappears in chemical transformations.

#### 2.2.1. Mechanochemical Transformation in Mortar

Thousands of years have passed from the first inorganic synthesis in a mortar, the cinnabar conversion to mercury in a copper mortar [78], to the first organic synthesis by milling. After the mechanochemical synthesis of tetrachloroquinhydrone [79], silence reigned for nearly a hundred years [80]. In both cases, the prepared charge-transfer complexes are co-crystals, and the process did not lead to the formation of covalent bonds. These attempts were the first steps in organic mechanochemistry. After that, it took another 30 years or so for the first mortar and pestle actual organic mechanosynthesis. Although the preparation of phenylaldoxime from cavitands only appeared about a decade ago [81], this simple synthetic method has now begun to penetrate educational laboratory practice [82]. Albeit the synthesized cavitand is somewhat similar to cyclodextrins, reports on the production of CD derivatives in mortars have not appeared in recent decades.

Mortar and pestle manipulation of αCD and bis(3-aminopropyl)-terminated polytetrahydrofuran resulted in a complex, a pseudorotaxane, in which the terminal amines reacted with 3,5-dinitrobenzoyl chloride or 2,4-dinitrofluorobenzene in a mortar, too, providing a rotaxane after one-hour grinding. Both reactions use mortar, unlike the PEG and polytetrahydrofuran with allyl ether end cappings, where an N-oxide converted the pseudorotaxanes of native and permethylated αCDs to the rotaxane derivatives. In the latter case, the preparation of the pseudorotaxane used US, which is also a green technique.

#### 2.2.2. Mechanochemical Transformation in Mills

As described earlier, BMs are suitable for the preparation of CD complexes. The advent of high-speed or high-energy BM variants has radically changed and expanded the spectrum of synthetic possibilities. Today, these devices are capable of producing up to kilograms of compounds. Not all the BM-types are suitable for all reactions, and the experimental conditions are not always comparable. In the so-called Finkelstein reaction, the benzyl chloride conversion to benzyl, a better conversion was achieved in a significantly shorter grinding time in a planetary than vibrating mill [16].

The limited solubility of CD intermediates in various solvents often makes the CD-derivatizations laborious, and sometimes the reagent’s solvent incompatibility also poses a challenge to chemists. The potentially contrasting solubility profiles of the starting and target CD derivatives can often complicate syntheses, too. Frequently, but not always, some high-boiling solvents can be a reasonable compromise, but difficulties in removing the solvents, reagent residuals, or byproducts make production expensive or environmentally unsuitable. In many cases, the syntheses, except the easiest-to-produce CD derivatives, are only possible in DMF or DMSO, which has high energy demand to remove. For CD derivatives prepared in aqueous media, energy-intensive water removal is also necessary. Water can also be very reactive under certain reaction conditions, primarily by hydrolyzing the reagents, which complicates the preparation of many CD derivatives, as HPβCD or SBβCD.

A good example is the preparation of HPβCD. This reaction occurs in an aqueous solution in the presence of a strong base, usually NaOH. In a BM reaction, the much smaller amount of water significantly decreases the hydrolysis, and the better utilization of the reagent also allows a reduction in the molar amount of the reagent. As long as the DS of the product is low, HPβCD can be “crystallized” from acetone, which can reduce the propylene glycol to an acceptable level. Acetone, while not exactly a green solvent, is at least easily regenerated. At a higher DS, the removal of propylene glycol can be a complication, and a multistep synthesis is necessary [83]. In a BM reaction, due to the much smaller amount of water, the hydrolysis is significantly suppressed; furthermore, the better utilization of the reagent also allows for the reduction of the molar amount of the reagent. The aqueous solubility of the epoxides used in CD derivatizations is low, so in anhydrous media, the contact area in BM is increasing [84]. This method enables the preparation of novel lipidyl CDs, which were previously much more difficult or impossible to prepare, and the reproducible preparation of polymerized CDs, too [85].

The *p*-toluenesulfonic acid derivatives are proper guest molecules for βCD, and the orientation of the sulfonyl group depends on many factors. In water, the continuous exchange of the host considerably reduces the secondary hydroxyl substitution. In solids, on the other hand, the orientation of the chlorosulfonyl group is fixed in the βCD cavity after complexation for geometrical reasons. Thus, when the chlorosulfonic acid group is in spatial proximity to the secondary alcohol groups, this allows selective derivatization of secondary hydroxyls without protecting the primary ones. The reaction center is usually O(2) because of their more favorable position than the O(3) groups. In the presence of a strong base, a mannoepoxide formed immediately from the O(2) tosyl ester [86]. Although in BM, the complex formation with the oppositely oriented tosyl group also occurs, the primary tosylation is minimal. This reaction can also provide indirect evidence for the greater reactivity of O(2), as has previously been deduced from the substitution pattern of hydroxyalkylation and methylation reactions. The use of 6-periodo CDs in the exchange reaction of the halogens → 3-carboxyethylthio moieties is less favorable than the per-6-bromo CDs, which worked not only faster but also had a higher yield [17]. A similar effect exists in the halogen–azide exchange, too. When the NaI complex did not affect a further reaction, as in the monosubstitution, minimal reaction rate differences were in the halogen (or tosyl) groups. In the 6-persubstituted case, the formed iodide complex can leave the cavity only by a slow diffusion, which reduces the rate of further reactions. Although the worst guest among the alkali salts, the chloride salts, would be the most suitable 6-perhalogeno derivatives (the fluorides are inappropriate for a nucleophilic exchange), the reduced reactivity of per-6-chloro CDs did not allow a productive reaction [17].

Random substitution, although broadly following the reactivity trend observed in the solution reactions, resulted in more primary hydroxyl-side substitution. Because of the limited diffusion, not surprisingly, the reactive species is reacting the closest appropriate hydroxyl while in solution, the reagent has more time to find the most reactive partner [18].

Only a few kinetic studies have appeared in the short history of the CD-related BM reactions. In vibrating BM, the reaction rate between mesitylenesulfonyl chloride and CDs showed initial particle dependency [87], which lacks in the case of rotational BMs. The production of transition metal/CD composites takes advantage of the higher comminution effectiveness of rotary BMs compared to vibrating versions [88]. The water content of the components in both types of BMs can cause difficulties, as the warming of the ground solid can result in phase transitions. These can transform the powders into rocky or sticky solid cores, lumps, or glassy materials [24,89]. Clumping can—but not always—disappear as the reaction proceeds [85]. Dehydration of the CDs or an inert liquid additive can reduce these unwanted side effects, and finally, even mechanical intervention by the operator can break up the initially formed hard materials [90].

BM reactions are suitable for the synthesis of SiO_2_/CD composites [91] and chemically stable CD polymers [85,92]. The water and pH more sensitive CD–nanosponge preparations are also an excellent target of the mechanochemical syntheses. Classical nanosponge syntheses also use high-boiling solvents [93,94]. The potential pharmaceutical applications require removal not only of the solvent but also of unreacted and degraded reagents. The suppressed decomposition of the reagents makes the workup of the nanosponge crude products prepared in BM simple, and the filtration of the water-insoluble residual reagents can provide high purity products [95].

#### 2.2.3. Mechanochemical Transformation Using Ultra-Turrax

As ultra-turrax works well in homogenization, solubilization, disintegration, and complexation, its use in chemical reactions has a technical limit. The design of the devices does not allow a continuous long-period operation, and since most organic reactions are not instantaneous, the use of these devices for synthetic purposes is severely limited. Even though the more effective energy-transfer green methods use 30–60 min reactions, the fast-stirring-triggered reactions are rare. The ultra-turrax-assisted preparation usually results in non-covalent associations between CDs or CD complexes and matrix. These composites can be stable for a long time and suitable for controlled release of the complexed drug. The preparation of tuned CD-contained hydrogels is possible by high-speed mixing technology [96], but these are not chemical reactions in the classic meanings.

#### 2.2.4. Mechanochemical Transformation Using Ultrasound

Sonication, such as the ultra-turrax, is very popular in hydrogel preparation, which rarely results in stable, covalently bound CD derivatives [97,98]. Without reading the article, it is usually hard to deduce whether the topic is about a verbatim sonochemical reaction or solution preparation, homogenization, and eventually complexation. In many times, the technical papers refer to the formation of non-covalent interactions, H-bonds, or ionic associations as syntheses or derivatizations also. Obviously, not all metal ion–CD interactions are stable or of a specific composition enough to function as compounds in various applications. Although sonication in dissolution, crystallization, or homogenization has a long history, the application of US in CD derivatization by chemical reactions is relatively new [99]. A complicated mechanism of complexation and zinc promoted dehydrohalogenation of glycosyl bromides provided glycals [100]; however, the role of βCD and the reaction mechanism is not entirely understood. Alkaline earth metal oxides and hydroxides are strong enough to ionize the secondary alcohols of CDs and suitable to form stable complex 3D structures with suitable inorganic salts for analytical applications [101]. Another example of the utilization of stable salt formation is the preparation of the copper nanoparticles. The synthesis of microporous Cu(I) or Cu(0) solids [102,103,104] also uses sonication, exploiting the salt formation of Cu^2+^ to βCD [105] as a template. US irradiation provided a stable Si-O-CD compound copper catalyst with enhanced CD and Cu content [106].

Silver doped nanosponges and magnetic SiO_2_/CD hollow spheres showed limited stability under US irradiation and demonstrated restricted recyclability caused by acoustic cavitation. In contrast, zinc peroxide and citric acid/βCD adduct sonication produced a composite that was more stable and effective in hydrogen peroxide decomposition than the ZnO_2_ alone [107]. A magnetic nanoparticle of CD complex preparation is an example of the non-conventional CD complexes; that is, in this case, the 6-monoamino-βCD is the ligand which forms a stable complex with magnetic particles in a deep eutectic solvent [108]. Amino acid–modified βCD can form a stable association with CdSe/CdS quantum dots after sonication in a hexane/water emulsion [44,46,109]. Many publications appear on the metal–organic networks which report their synthesis with ultrasound participation. Although these composites can be stable under various conditions, they form a transition between the complexes and covalently or ionic bound components. A recent review on the preparation methods of various metal–organic networks summarizes the state-of-the-art knowledge, and these composites are out of the scope of the present paper [110].

In situ preparation of permethylated αCD polyrotaxanes from the initially prepared PEG pseudorotaxanes was effectively carried out by sonication [111].

The synthesis of various per-2,3-*O*-alkyl-6-TBDMS-βCDs was carried out in DMF, using US [112,113]. These fluidizable derivatives are suitable for coating glass surfaces and showed excellent chiral gas-chromatographic separation properties. The best utilization of the US-assisted synthesis is when an activated CD derivative reacts in a neat liquid reagent. The classic preparation method uses dry DMF with a couple of molar excess (to the CD) of the reagent, which is very effective in monosubstitution cases, but when a multi-substitution has aimed the traces of dimethylamine, a decomposition product of the DMF, it inevitably forms contaminates. In these cases, the high mole ratios of the required amines allow their use as solvents. The poor solubility of these activated CDs can be overcome by sonication, as was performed in the synthesis of per-6-alkylamino βCDs. Even though the non-conventional reaction conditions, MW or US irradiation, did not improve the yield in the reaction of βCD and methyl (3-bromopropyl)-2-iodobenzoate, the reduction of the long reaction time was successful, from three days to 4 h (US) and 1 h (MW) [114]. The 6-monotosylation of CDs in water is always a challenge because of the reagent and product instabilities. The hydrolysis of the activated tosyl reagent can reduce the yields, while in the case of α-and γCDs, the reaction is unsuccessful. Use of US in a more concentrated solution of CDs and tosyl imidazole resulted in 6-monotosylated α/β/γCDs in good yield [115]. The use of US in a more concentrated solution of CDs and tosyl imidazole resulted in 6-monotosylated α/β/γCDs in good yield. A slight modification in the reaction conditions resulted in 2-monotosylated CDs, of which further conversion gave mono-altro-azido analog of the CDs. A combination of US and MW activation of the copper powder and IRIS 3 and IRIS5 cyanine dyes successively reacted with 6-monoazido-βCD in 1,3-dipolar cycloadditions [116].

A nanosponge synthesis used high-temperature (90 °C) sonication to get unified solid particles; however, the purification used Soxhlet extraction, which reduces the green value of the synthetic method [117,118]. Hexamethylene diisocyanate crosslinked βCD nanosponge in warm DMF was prepared in high yield and had an almost three-times-higher BET surface area than without sonication. The US treatment does not always result in significant reaction acceleration, as seen in the 6-monoazido-βCD conversion by Pd/H_2_ to the monoamino derivative [119], compared with the classic transfer hydrogenation method [120].

Ultrasound-triggered transesterification of methotrexate–acylglycerols and methotrexate–α/β/γCD conjugates by immobilized esterase resulted in multiple methotrexate substituents on the CDs [121].

Although no chemical reaction occurred, US treatment resulted in nanoparticles of insoluble βCD polymer in organic solvent mixtures, for which gel was suitable for coating capillaries for chiral gas chromatography [122].

#### 2.2.5. Mechanochemical Transformation Using Hydrodynamic Cavitation

In many cases, acoustic cavitation is also called hydrodynamic cavitation, despite the different backgrounds. Unlike the flowing systems, the US waves do not have translational movement in an ideal case. To our best knowledge, no publication on the use of hydrodynamic cavitation has been registered in the major literature databases on the synthesis of CD derivatives or destruction of the macrocycle by it.

#### 2.2.6. Mechanochemical Transformation Utilizes Shearing

Shearing can occur in any mechanochemical manipulation and is usually cumbersome to study on its own. Although the synthetic utilization of the effect alone is difficult to exploit, many times the controlled release of a drug substance or, as mentioned in Section 2.1.4, the prepared cell-targeting magnetic CD nanoparticles are suitable to destruct cancer cells by shear triggered decomposition of macromolecules.

#### 2.2.7. Mechanochemical Transformation Using Tribology

Mechanochemical transformation modified tribological properties of iron surfaces in an aqueous PEG 600 solution with βCD/dialkyl pentasulfide (DPS) inclusion complex. The complex showed better tribological properties than βCD alone and better anti-friction properties than DPS alone. The βCD molecule decomposed into different molecular fragments, which released DPS molecules under friction conditions. The iron sulfide films formed from DPS and iron surface resulted in the creation of an anti-friction property at the FeS–FeS interface. In this process, the mechanochemical transformation decomposed the cyclodextrin, which resulted in the release of the guest molecule, and finally led to the reaction between the released DPS and iron [123].

## 3. Conclusions

Mechanochemistry is a constantly and almost exponentially evolving field with many green aspects. There is still little information available on macrocyclic mechanochemistry, and although many chemists have used mechanochemical manipulations for a long time, most of them are rarely directed consciously at exploiting the benefits of mechanochemistry. Any mechanochemical manipulations constitute different micro-processes, and it is impossible to create a clean environment controlled by a single process. Energy transfer also varies over a wide range, which significantly affects the behavior of the studied system. In cyclodextrin chemistry, mechanochemical methods are predominantly for particle size reduction, homogenization, or complexation. In a traditional sense, the formation or modification of non-covalent interactions is not a true chemical transformation.

The most popular technique is the application of ultrasound for a complex preparation, whether it is a CD complex, injection of a CD into a matrix, or the CD dispersion on the surface of a metal compound, or the preparation of metal–organic networks. US-assisted chemical transformation is usually, but not always, faster than classical reactions and yet often suffers from the use of solvents.

In terms of energy efficiency, solid-state transformations appear to be the most eco-friendly technology due to the significantly reduced amounts of solvent used. The limited mobility and degradability of components in solid-state reactions can open up an efficient synthetic route. Sometimes, it also allows the synthesis of CD derivatives that is too complicated to achieve by conventional ways, if possible at all. It should also keep in mind that the production of starting materials for these syntheses is often far from the requirements of green chemistry. Although the various mechanochemical transformations are useful tools, and as with all processes, they are not universally applicable. Recent and emerging research could significantly expand the greener transformations in CD derivatization.

## Abbreviations

Ball mill	BM
Cyclodextrin	CD
Dialkyl pentasulfide	DPS
Dimethyl sulfoxide	DMSO
(2-Hydroxy)propyl-βCD	HPβCD
MW	Microwave
N,N-Dimethyl formamide	DMF
Nanoparticle	NP
Polyethylene glycol	PEG
4-Sulfobutyl-βCD	SBβCD
Three-dimensional	3D
Tris(hydroxymethyl)amine	TRIS
Ultrasound	US
